# Comparing plasma levels and ratios of tryptophan, serotonin, kynurenine and quinolinic acid between patients with bipolar disorder and healthy controls: impact of depressive episode or post-traumatic stress disorder symptoms

**DOI:** 10.3389/fpsyt.2025.1680907

**Published:** 2026-01-12

**Authors:** Valerio Dell’Oste, Lionella Palego, Andrea Bordacchini, Berenice Rimoldi, Livia Parrini, Virginia Pedrinelli, Gino Giannaccini, Laura Betti, Claudia Carmassi

**Affiliations:** 1 Department of Clinical and Experimental Medicine, University of Pisa, Pisa, Italy; 2 Department of Mental Health and Addiction, Azienda USL Toscana Nord-Ovest, Lucca, Italy; 3 Department of Pharmacy, University of Pisa, Pisa, Italy; 4 Department of Mental Health and Addiction, Azienda USL Toscana Nord-Ovest, Livorno, Italy

**Keywords:** bipolar disorder, depressive episode, kynurenine, plasma, post-traumatic stress disorder, quinolinic acid, serotonin, tryptophan

## Abstract

**Background:**

Bipolar disorder (BD) is a chronic mental illness displaying recurrent episodes of impaired mood, in alternance with euthymic or subsyndromal periods. The comorbidity between BD and post-traumatic stress disorder (PTSD) is attracting attention due to its frequency, diagnostic difficulties, and worsening prognosis. Consequently, identifying molecular substrates of post-traumatic vs. depressive symptoms in BD would provide much benefit for patients’ rescue. This study thus focused on tryptophan (TRP) metabolism, at the crossroad between neurotransmission, immunity and inflammation, under distinct BD mental conditions.

**Methods:**

We compared plasma TRP, serotonin (5-HT), kynurenine (KYN) and quinolinic acid (QUIN) among 20 euthymic-BD patients with PTSD (PTSD group) or 20 with depressive episode (DEP group) and 24 controls (CTL group). Metabolic ratios were calculated to monitor TRP-path fluxes. All participants underwent clinical examinations by psychometric instruments as the: Structured Clinical Interview for DSM-5 disorders (SCID-5), Hamilton Depression Rating Scale (HAM-D), Young Mania Rating Scale (YMRS), Impact of Event Scale-Revised (IES-R), Mood Spectrum-Self-Report lifetime version (MOODS-SR), Trauma and Loss Spectrum-Self Report lifetime version (TALS-SR), Work and Social Adjustment Scale (WSAS). Blood withdraws were also achieved, followed by plasma measurements through ELISA.

**Results:**

Both DEP and PTSD-groups revealed markedly lower 5-HT and 5-HT/TRP values than controls, with additionally decreased 5-HT in DEP- vs. PTSD-subjects. DEP-patients also showed reduced TRP and increased QUIN, KYN and QUIN ratios vs controls, while PTSD-subjects displaying only increased QUIN/KYN values. Additionally, several correlations between biochemical and clinical parameters were reported. Noteworthy, 5-HT and 5-HT/TRP were negatively correlated with all psychometric measures, while all biochemical parameters studied, except KYN, were correlated with poor socio-personal functioning; otherwise, KYN-shunt parameters better distinguished the severity of DEP symptoms than the PTSD ones.

**Conclusions:**

Substantially, BD-patients with low 5-HT and severe burden could be distinguished based on KYN profiles/trajectories when affected by depression or PTSD, entailing different neuroinflammatory/neuroendocrine patterns in mood vs. post-traumatic stress dimensions. This will encourage deeper assessment of BD metabolic/inflammatory biomarkers, allowing for refined patients’ stratifications and targeted therapeutic interventions.

## 1 Introduction

Bipolar disorder (BD) is one of the most debilitating mental illnesses, characterized by recurrence of mood episodes (manic/hypomanic, depressive), poor quality of life, elevated individual/social disability, and pronounced risk of suicide (1). The global burden of this condition on health is quite worrying: indeed, its lifetime prevalence is about 2% worldwide (2), with about 40–50 million people currently affected corresponding to an incidence of about 0.53% (1). Moreover, BD represents one of the major challenges in clinical psychiatry due to the objective difficulty at attaining patients’ favorable outcomes and the high heterogeneity of the illness, defined since decades (3), both among affected individuals and within them. BD is classified into type I, type II, Cyclothymic disorder and other drug- and medical-induced or non-specified conditions (4). The BD complexity also derives from the commonly encountered comorbidity with other mental disorders, such as anxiety disorders as well as with trauma-related disturbances and post-traumatic stress disorder (PTSD) (5, 6). In the clinical practice post-traumatic spectrum symptoms or full-blown PTSD are frequently diagnosed in BD of both type I or II (7–10), resulting in confusing and overlapping conditions that can be overlooked or misunderstood, increasing the risks of unsatisfactory responses to treatments, relapses, tendency to commit suicide, and/or develop new and more severe symptom profiles (7, 11). It is important also to highlight that PTSD can occur in euthymic bipolar patients, suggesting a background susceptibility towards posttraumatic stress in this population, even when mood is under a proper therapeutic control (12). Furthermore, patients with BD exhibit behaviors that may predispose them to traumatic experiences.

PTSD is a mental disorder that has been more recently identified with respect to BD, constituting an area of investigation that has not yet been fully explored (13). Symptoms of PTSD may occur after exposure to severe trauma, e.g. wars, terroristic attacks, genocides, natural disasters, pandemic emergencies, sudden or violent death of loved ones, captivity, personal aggressions, abuse or discrimination in one’s life. PTSD represents a highly disabling mental condition: its peculiar symptomatology is defined by prolonged and intense psychological suffering which often results in chronic and unfavorable progressions (14, 15). The up-to-date main diagnostic criteria for PTSD describe a variety of symptom clusters grouped into 4 main dimensions, which include, respectively: intrusions of negative thinking and re-experiencing perceptions, avoidance behaviors, negative mood and cognition, as well as impairments in arousal and reactivity to daily stimuli (DSM-5-TR, 4). The lifetime prevalence of PTSD varies across studies upon the clinical instruments adopted for the assessments, and it is estimated around 3.9% in the worldwide with double rates in female rather than male subjects (10–12% vs. 5–6%) (16). Thus, currently, PTSD represents a focal point in the mental illness landscape, both because of its frequency and the still poorly understood and countered negative impact it can have on public health. PTSD high heterogeneity and multifaceted presentation have led clinicians to appraise this condition within a spectrum of signs and traits, by using psychometric examination tools such as the “Trauma and Loss Spectrum” questionnaire (17). Such peculiar vulnerabilities inherent to the post-traumatic stress dimension are therefore paramount aspects for attaining an adequate follow up, monitoring and prevention from the worsening of patients’ clinical pictures (18). Another foremost aspect to consider in this framework is the fact that the typical PTSD symptomatology, particularly the presence of protracted negative perceptions, avoidance/numbing, re-experiencing of the trauma and hyperarousal, can significantly affect sleep physiological phases, resulting in disrupted circadian rhythms and non-refreshing slumber (19). These peculiarities have been found related to the elevated propensity of PTSD patients in developing somatic and neurologic symptoms (20). Among the most reported somatic consequences in PTSD there are pain-related functional disturbances and chronic fatigue (21, 22), cardiometabolic diseases (23, 24) and even neurodegeneration (25), causing a severe burden for the quality of life of affected people, their families and for the whole community. In the presence of concomitant BD and PTSD, it is expected that these vulnerabilities may even be additive, considering that currently available pharmacological therapies for patients with more severe PTSD, as well as for individuals with BD in comorbidity, are still unspecific, making biological and pharmacological research in the field urgently needed (26). Under these perspectives, translational psychiatry investigations, aimed at obtaining non-invasive peripheral biomarkers, are considered helpful strategies for clinicians to distinguish the multiple PTSD facets, to stratify and describe the various phenotypes, allowing for more specific, effective, and possibly individualized therapeutic interventions (27–29).

Despite the still scarce investigation conducted so far, as well as the little and relative knowledge in this area, several authors have evidenced that PTSD patients may display altered physiological responses to stress, precisely disturbed and unbalanced sympathetic “fight-or-flight” cascades vs. the parasympathetic “rest-and-digest” ones, involving the cortisol-releasing hypothalamic-pituitary-adrenal (HPA) axis together neuroimmune/inflammatory activities. Such alterations would be mirrored, for instance, by the increased release of pro-inflammatory cytokines into the bloodstream as well as by changes of the redox buffering molecular arsenal (30–32).

In the search of biological profiles and signatures of these dysfunctions, a well-known metabolic pathway, related to the biotransformation of the essential amino acid tryptophan (TRP), is attracting increasing interest (30, 33). Tryptophan (TRP) is a large neutral amino acid (LNAA) that contains an indole ring as the -R substituent, a distinct chemical structure among all other amino acids preserved in the non-protein metabolic pathways that give rise to the mood neurotransmitter serotonin (5-HT), the pineal circadian hormone melatonin (MLT), as well as trace tryptamines exerting neuromodulation in the CNS and immunomodulatory actions in the periphery (34, 35). In addition, it must be highlighted that these biomolecules have been already found impaired in adaptive disorders and even PTSD, both in patients and animal models (36–39). The interest on investigating TRP biotransformation in the body is also related to the fact that the main non-protein metabolic route of this amino acid is not the 5-HT- and MLT-generating one, which transform only the 1-5% of its total amounts, but the so-called kynurenine (KYN) shunt or TRP catabolic path (TRYCAT). The KYN shunt gives rise to a variety of small molecules at the crossroad between immune and neuroendocrine mechanisms, particularly those implicated in molecular processes of stress coping disrupted in PTSD (30, 40–42). As a matter of fact, the KYN metabolism has been linked to significant changes in “fight-or-flight” and immune-inflammatory responses, energy metabolism and increase of oxidative stress (43–45). The KYN-shunt starts by metabolizing TRP into N-formyl KYN by the ubiquitous enzyme indole-2,3-dioxygenase (IDO), present in two distinct isoforms IDO-1 and IDO-2, or, to a lesser extent and mainly in the liver and kidney, by the enzyme TRP-2,3-dioxygenase (TDO). The main KYN-shunt initiating enzymes IDO and TDO differ not only for their localization, but also for their substrate selectivity and physiological role: TDO, expressed overall in the liver, recognizes TRP only, being directly involved in the homeostasis of this amino acid and in protein synthesis under the control of cortisol and HPA activity; conversely, IDO is overall active in the immune system under the regulation of cytokines and other immune-inflammatory molecules (46). This metabolic pathway continues through the production of KYN, which in turn further cleaves into several possible branches, generating anthranilic acid (AA), xanthurenic acid (XA) or kynurenic acid (KYNA), quinolinic acid (QUIN) or picolinic acid (PA) (35, 47). The QUIN branch of the KYN shunt is also considered the “energy metabolism” one, as it leads to the formation of NAD^+^ and branches further, upstream of the QUIN formation, into a different pathway generating acetyl-CoA (35). Moreover, the QUIN path is also related to epigenetic mechanisms, due to the NAD^+^ involvement in sirtuin activation and histone deacetylation (48). The QUIN formation route from KYN is also known as the CNS “excitogenic” one: QUIN is indeed a partial agonist of the NMDA kainate and α-7 nicotinic acetyl choline receptors (45, 49). Thus, QUIN is also a pro-oxidant compound, potentially “neurotoxic”, since its accumulation inside the CNS can result in hyperexcitation, increase of reactive oxygen species (ROS), and apoptosis (35). For this reason, QUIN increase in cells can happen only transiently under specific circumstances, not only in neurons but also, for instance, in hepatocytes (50): under physiological conditions, TRP catabolism goes mainly toward ATP formation and only a minor portion leads to QUIN or NAD^+^ accrual (51). Another core KYN route generates instead KYNA by the action of the enzyme KYN aminotransferase (KAT), a KYN branch considered as the “neuroprotective” one, since this intermediate is an antagonist of central NMDA receptors (44, 45). These trajectories afford evidence that the KYN shunt plays an important “balancing” role between excitatory and inhibitory processes in the CNS and in the whole organism, which acquires relevance in particular metabolic necessities, energy production adaptations (52) and allostasis load conditions (34, 53).

For instance, the generation of KYN and its metabolites is significant during development and immune system maturation where selective cell survival is crucial and increased energy metabolism is required, or, as well, in the context of intense defence responses, when lymphocyte clonal differentiation as well as shortages/unavailability of active biomolecules occur, due to their engagement under strong distress (45, 54). It has been also reported that genetic defects of the enzyme IDO-1 are associated with autoimmune diseases, implying a main role of this enzyme in immune tolerance (55). Essentially, the QUIN and KYNA branches of the KYN shunt are dynamically and reciprocally adapted during cell differentiation processes (45, 54) to avoid remarkable accrual or loss of these two substrates over time. Among the close cross-relationships existing between the KYN metabolic fluxes, neuroinflammation and immune/inflammatory responses, there is the activation of the limiting enzyme IDO by the release of several cytokines, such as IL-6, IL-1β or TNF-α (44, 52). Therefore, even the overstimulation of IDO, due to the persistence of an inflammatory state, can promote pathogenic mechanisms, giving rise to a hyperactive/dysfunctional KYN pathway to the point of significantly varying the circulating levels of its metabolites and in particular of QUIN, in the context of an unbalanced regulation of the NAD+ and acetyl-CoA branches.

**Figure 1:**
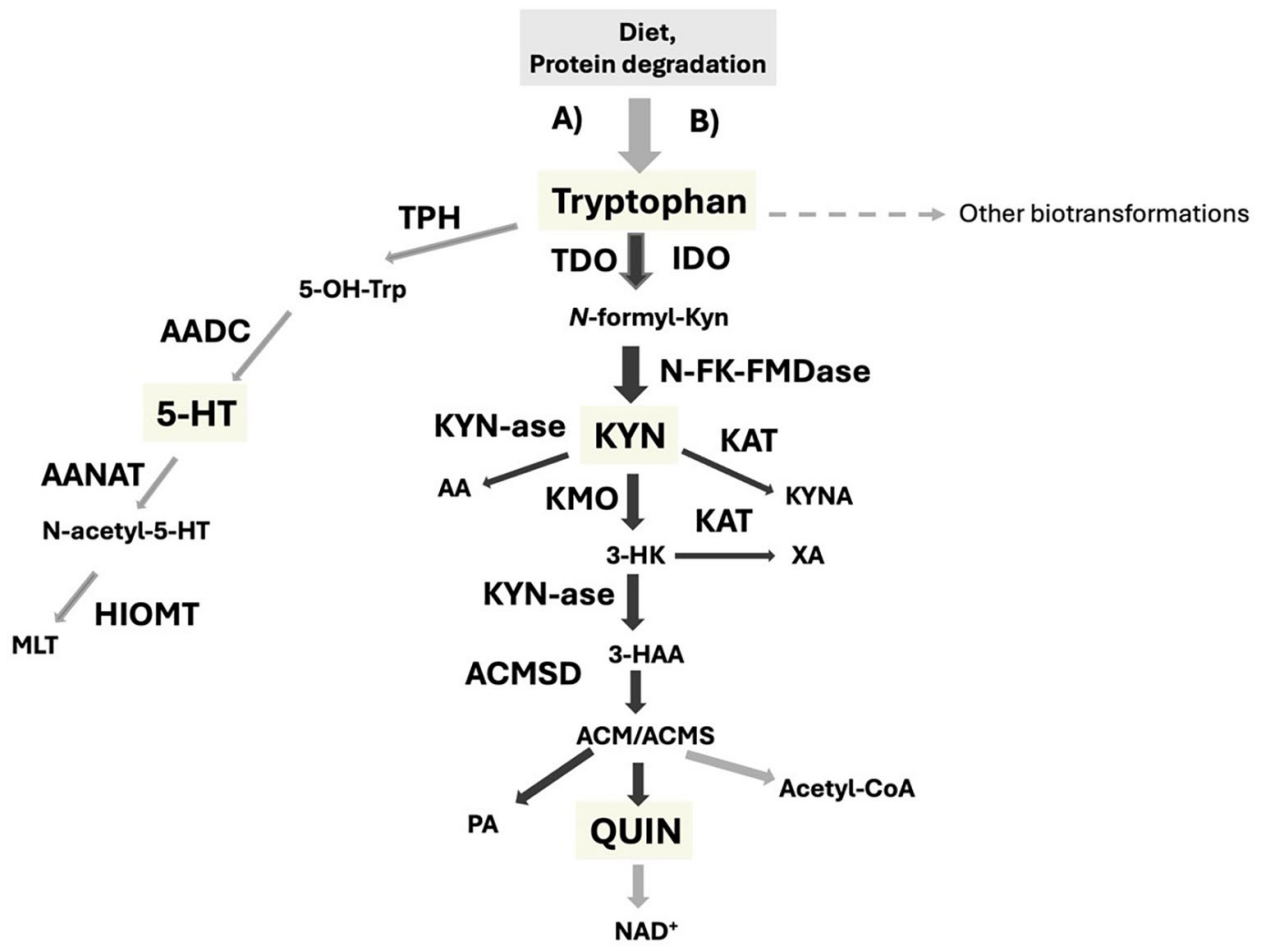
The two main non-protein metabolic pathways of the essential amino acid tryptophan in humans, **(A)** the indole conserving pathway generating serotonin, melatonin and tryptamines; **(B)** the kynurenine shunt: the indole disrupting pathway. Other biotransformations indicate other less known indole-related metabolic pathways. Substrates: 5-OH-TRP: 5-hydroxy-tryptophan;5-HT: serotonin; N-acetyl-5-HT: N-acetyl-serotonin; MLT: melatonin;N-formyl-KYN: N-formyl-kynurenine; KYN: Kynurenine; AA: anthranilic acid; KYNA: Kynurenic acid; 3-HK: 3-hydroxy-kynurenine; XA: xanthurenic acid; 3-HAA: 3-hydroxyanthranilic acid; ACM: 3-amino-3-carboxy muconic acid; ACMS: 3-amino-3-carboxy muconic acid semi-aldeyde; PA: picolinic acid; QUIN: quinolinic acid. Enzymes: TPH: Tryptophan hydroxylase (EC 1.14.16.4); AADC: Aromatic-amino acid decarboxylase (EC 4.1.1.28); AANAT: Arylalkylamine N-acetyltransferase (EC 2.3.1.87); HIOMT: Hydroxy-indole-O-methyl transferase HIOMT, (EC 2.1.1.4); TDO: Tryptophan 2,3 dioxygenase (EC 1.13.11.11); IDO: Indole 2,3 dioxygenase (EC 1.13.11.52); N-FK-FMDase: N-formyl-kynurenine formamidase (EC 3.5.1.9); KAT: Kynurenine aminotransferase (EC 2.6.1.7); KYNase: Kynunerinase (EC 3.7.1.3); KMO: Kynurenine monooxygenase (EC 1.14.13.9); ACMSD: 3-amino-3-carboxymuconic acid semialdeyde decarboxylase (EC 4.1.1.45).

It is not surprising that investigations on KYN pathway are considered by some scientists among the most important ones to unveil biochemical signatures of different endocrine/metabolic distress conditions in psychiatric disorders (56). Unbalanced TRP degradation across the KYN pathway, accompanied by the release of potentially neurotoxic metabolites, has been already implicated in many mental illnesses (45, 49, 51). Interestingly, lower peripheral levels of TRP and KYN or KYN metabolites were reported in meta-analyses on mood disorders and schizophrenia (57–59). Furthermore, a higher production of QUIN with respect to KYNA has been rather found in mood disorders than schizophrenia, suggesting its use as a biomarker of mood alterations rather than psychosis (59). In more detail, a lower KYNA/QUIN ratio has been found typically linked to mood disorders, while lower KYNA/KYN and increased KYN/TRP ratios have been detected more specifically in both schizophrenia and major depressive disorder (58–60). It is noteworthy that increased QUIN and KYN levels have been associated with suicidal ideation/behaviors (61–64). In this context, it appears pertinent to evaluate circulating amounts of 5-HT, deriving from the “opposite” arm of TRP metabolism, simultaneously with the levels of KYN metabolites, not only because of the key action of this monoamine as a mood adaptor and modulator of inflammation, but also to better understand the communication dynamics between these two opposing pathways of biotransformation of the initiating substrate, TRP. In **Figure 1** is reported a simplified schema of these two main branches of the non-protein TRP metabolism in humans, also showing their interplays with energy metabolism (KYN paths), neurotransmission/inflammation (5-HT and KYN path in opposite direction) and circadian pacemakers (MLT). It follows that the relative measurement of TRP, KYN shunt substrates, and 5-HT in the plasma of psychiatric patients may be useful for distinguishing specific molecular patterns in different conditions.

Therefore, due to the lack of research on TRP metabolism in PTSD, especially in patients with comorbid BD (30), the aim of the present study was to fill a gap in the literature by precisely assessing bipolar patients with stabilized mood and PTSD symptoms, as a starting point to possibly reach an accurate stratification into more specific and even more complex BD phenotypes. In detail, we investigated herein the metabolic balance between the TRP-deriving indoles and the KYN routes, through the measure of the circulating concentrations of TRP, 5-HT, KYN and QUIN and their ratios, in a group of bipolar subjects in the euthymic phase showing PTSD symptoms (PTSD group) compared with a group of bipolar subjects under a major depressive episode (DEP group) without comorbid PTSD, and a healthy control group (CTL group). Essentially, we appraised the relative TRP metabolic flux producing the mood modulator 5-HT, in respect to the excitogenic and “hyperreactive” QUIN counterpart. Possible differences are suspected since PTSD symptoms have been found less sensitive to the effect of standard serotonergic antidepressant drugs in respect to depressive ones (65, 66).

Meantime, we also aimed at finding possible correlations among the amounts of these bloodstream molecules and specific clusters of symptoms in DEP and PTSD subgroups of patients.

## 2 Methods

### 2.1 Subjects

Patients with BD were recruited at the Psychiatry Unit of the Department of Clinical and Experimental Medicine of the University-Hospital of Pisa (AOUP), Pisa, Italy. Adult out- or in-patients were evaluated, selected and admitted to the study from those in a current euthymic phase with a PTSD diagnosis (PTSD group) as well as those with a current major depressive episode diagnosis (DEP group) without PTSD. Voluntary subjects, in good mental and physical condition, were also evaluated and recruited from a pool of healthcare professionals at the hospital as the control group for this survey (healthy controls, CTL group). Inclusion criteria for patients were as follows: age between 18 and 65 years; history and diagnosis of BD, according to the classification criteria of the DSM-5-TR, in the euthymic phase (only for the PTSD group) or experiencing a major depressive episode (only for the DEP group); history and diagnosis of current PTSD according to the classification criteria of the DSM-5-TR, at least one month after the traumatic exposure in order to exclude a possible acute stress disorder (only for the PTSD group); recruitment at the first visit or follow-up control for outpatients or the first day of hospitalization for inpatients, before any therapeutic intervention or variation in therapies applied at the time of clinical assistance and start of the study, thus comprising only subjects under a stable psychotropic treatment for at least one month; and acceptance of the protocol and signature of informed consent. Exclusion criteria for patients were as follows: age under 18 and over 65 years; history and diagnosis of relevant full-blown neurological or medical diseases, including inflammation-linked and autoimmune disorders; inability to sign an informed consent to the study; alcohol or substance abuse in the last six months; pregnancy; or a current diagnosis of acute stress disorder. Exclusion criteria for the CTL group (healthy volunteers) were the same for patients, except that the controls all had to be without a history of or current mental disease according to the DSM-5-TR. Trained psychiatrists assessed all recruited subjects by means of a structured clinical interview and the administration of suitable psychometric scales. Blood samples were also collected from all participants to appraise the plasma levels of TRP and its metabolites. All biochemical assays were realized at the Laboratory of Biochemistry of the Department of Pharmacy of the University of Pisa. All participants received clear information about the study and had the opportunity to ask questions before providing written informed consent. All data were treated according to Italian and European Privacy laws and rules. The study was conducted in accordance with the Declaration of Helsinki, and all procedures were approved by the local ethical committee (19299/21).

### 2.2 Psychometric instruments

Sociodemographic and clinical information was recorded in a case report form (CRF). Participants were investigated by means of the Structured Clinical Interview for DSM-5 disorders (SCID-5) to make a current psychiatric diagnosis, through the appraise of the presence of symptoms that may satisfy the specific diagnostic criteria (4, 67). Then, all recruited subjects were deeply examined by definite and validate psychometric tools to evaluate their mental state. These consisted in the most commonly questionnaires used in the clinical practice: the Hamilton Depression Rating Scale (HAM-D), a 21-item test that quantitatively assesses the actual severity of the depressive condition shown by the interviewed subjects with any possible change, taking into account both the extent of symptoms and their frequency (68); the Young Mania Rating Scale (YMRS), a 11-item scale that explores key symptoms of mania, generally present throughout the course, from the most modest symptoms to the more serious ones, to appraise the actual severity of the manic symptoms in the past 48 h, also based on the observation of his/her behavior by the doctor during the interview (69); the Impact of Event Scale-Revised (IES-R), a 22-item scale which assesses the actual severity of post-traumatic stress symptoms by measuring three core trauma features, which refer to the last week prior to the assessment (70).

Besides, both patients and controls were further investigated through validate lifetime dimensional/spectrum interviews. The primary aim of spectrum instruments is not to simply verify the presence or not of a mental disorder according to DSM-5-TR or other diagnostic tools, but rather to detect lifetime symptoms related to a certain psychiatric dimension, even at the sub-threshold level, by assessing its wide range of typical and atypical manifestations. Subthreshold and atypical symptoms, as well as personality characteristics, can co-occur with the full presentation of a psychiatric condition, as well as, intriguingly, in its absence (control population), albeit to a lesser degree. These instruments were thus developed according to a spectrum model of psychopathology, in the framework of the international research network called *Spectrum Project* (71–73).

In the present study, the following spectrum instrument were employed: the Mood Spectrum-Self-Report lifetime version (MOODS-SR), to appraise mood spectrum symptoms lifetime, in a dimensional perspective (74, 75); the Trauma and Loss Spectrum-Self Report lifetime version (TALS-SR), to investigate posttraumatic stress spectrum symptoms lifetime, in a dimensional perspective (17, 76). Briefly, the MOODS-SR investigates “mood,” “energy,” “cognition,” and “rhythmicity/vegetative” functions, by means of items organized into three manic and three depressive domains (74, 75); the TALS-SR is instead composed by 116 dichotomous questions (yes/no) grouped in nine key domains: loss events (I), grief reactions (II), potentially traumatic events (III), reactions to losses or upsetting events (IV), re-experiencing (V), avoidance and numbing (VI), maladaptive coping (VII), arousal (VIII), personal characteristics/risk factors (IX). TALS-SR is thus able, in the framework of a dimensional approach, to highlight the wide spectrum of trauma- and stress- related manifestations that may follow different kinds of stressful experiences across the lifetime (17, 77).

Finally, all subjects were also interviewed by the Work and Social Adjustment Scale (WSAS) to establish the global subjects’ functioning: WSAS is indeed a test used to evaluate and measure the individual work and social adjustment, by measuring the individual’s ability to perform the activities of everyday life and how these are modified in the week prior to the assessment by means of 5 items: the first investigates the work ability of the subject; the second one assesses the ability to cope with household tasks; the third item explores private recreational activities carried out by subjects; the fourth and fifth ones investigate the family interaction and relationship (78).

The interpretation of the entire battery of tests and questionnaires used here is based on indicating to clinicians that higher scores are obtained in the presence of more severe and marked psychiatric symptoms or dimensions, and, in the case of the HAM-D, YMRS and IES scales, on the application of cut-off values, specific to each scale, above which the symptom is no longer subthreshold.

### 2.3 Biochemical measurements

All blood samples were withdrawn, avoiding hemolysis, by the skilled and authorized nursing staff of the Psychiatry Unit of the Department of Clinical and Experimental Medicine University-Hospital of Pisa (AOUP), Pisa, Italy. About 15 mL of peripheral venous blood were collected from recruited subjects, who had been fasting from the previous evening and at least for 12 h. Blood withdrawals were carried out as part of routine blood tests between 9 and 10 a*.m*. to elude circadian rhythm interferences, according to the local ethical committee guidelines for this study. Briefly, blood was gathered in 3 vacutainer tubes (capacity = 3 mL each) containing K_3_EDTA as the anticoagulant, while 6 mL were added to a lithium heparin vacutainer (3 mL) and a clotting activator vacutainer (3 mL). Vacutainer tubes were then immediately transported in thermostatic containers by authorized medical personnel of the Psychiatry Unit to the Biochemistry Laboratory of the Department of Pharmacy, University of Pisa, related to the Clinical Psychiatry Unit for activities in the field of translational biomedicine, for sample preparation procedures, proper storage and analysis.

Blood was treated as previously reported (79, 80) to freshly obtain two different biological samples: platelet-poor plasma (PPP) and whole platelets. For the measurement of TRP, 5-HT and KYN, plasma (PPP) samples were employed, while QUIN was measured in both lithium-heparin plasma and serum (some subjects). Briefly, vacutainer tubes were always centrifuged within 30 min from withdrawal. All centrifugations were performed at room temperature (RT), 22–25 °C. The first centrifugation was conducted at low speed, 150 × g, for 15 min, to separate the platelet-rich plasma (PRP) from the other cellular elements. Then, the resulting PRP volume was transferred in a Falcon tube (capacity = 15 mL), carefully measured, precisely divided in half into two Falcon tubes and centrifuged at 2,000 × g for 15 min at RT, together the lithium-heparin and the clotting activator vacutainers. After this last centrifugation, the PRP yielded two different phases, collected as separate samples: the supernatant containing the K_3_EDTA-PPP and the whole-platelet pellets. In this study, only the PPP was investigated for the measure of TRP, 5-HT and KYN levels: EDTA-PPP was aliquoted in high-quality, low-binding protein Eppendorf Safe-Lock tubes (Sigma-Aldrich, St. Louis, MO, USA), and stored at -80 °C until assay, together lithium-heparin plasma and serum aliquots, these last used instead for the measurements of QUIN. The platelet pellets were stored at -80 °C and employed for other evaluations.

To measure the plasma concentrations of TRP, 5-HT, KYN and QUIN in our sample we used for each analyte a dedicated Enzyme-linked Immuno-sorbent Assay (ELISA) technique, modified for the accurate quantitative analysis of low-molecular weight substrates, consisting of commercially available indirect competitive ELISA kits, produced by ImmuSmol (Bordeaux, France). Immunoassays have been indicated as valuable and economical alternatives to the use of HPLC/U(H)PLC techniques in biological samples as plasma and others (81). In our experience, these techniques permitted suitable scheduling for subjects’ recruitment, blood withdrawals, biological sample treatment, storage and simultaneous analyses of samples from many patients and controls under the same condition. The provided technical instructions for each kit were accurately followed during the analysis. TRP, 5-HT and KYN were measured in thawed PPP samples, while QUIN was measured in thawed heparin-plasma and serum. All reagents and chemicals used for the study were of the best quality and purity, in agreement with the gold-standard guidelines of analytical laboratories. All required solutions were prepared using an ultrapure HPLC gradient-grade milli-Q water (18 MΩ cm, resistivity), obtained by means of a Simplicity Millipore Apparatus equipped with an UV lamp and a 0.2-micron filter to prevent contamination by particles and biological agents. The competitive ELISA assays were based upon two main analytical steps: a first step on which analytes were chemically derivatized and a subsequent step enabling the immune-enzyme determination of the derivatized analyte amounts. Each TRP, 5-HT, KYN and QUIN assay was provided with 6 ready-to-use standard solutions at known concentrations for the quantitative analysis: these ranged from 0 to 1,220 μM for TRP; from 0.015 to 2.5 ng/mL for 5-HT; from 0 to 10,000 ng/mL for KYN; from 0 to 2,032 ng/mL for QUIN. All kits provided a quality control system, consisting in a low- and a high-concentrated sample at known validated amounts, to check the entire procedure.

After derivatization, standards, samples and controls were transferred from the derivatization 96-well micro-plate to the second one, pre-coated with a same fixed amount of the derivatized analyte, to carry out the ELISA competition. The competitive reaction consisted in an overnight incubation at 4 °C, where derivatized analytes in standards, controls and samples contended with the pre-adhered derivatized analyte for a specific antibody which was also added in equal volume to all the microplate wells. After performing incubation and washing procedures, the detection reaction was started by adding a secondary antibody linked to the horseradish peroxidase (HRP) enzyme.

After the incubation with the detection system and further washing steps, the HRP substrate 3,3′,5,5′-Tetramethylbenzidine (TMB) was added avoiding light exposure and incubated for 25–30 min at 25 °C in a thermo-agitator (PST-60HL, Biosan, Riga, Lativia). This reaction was stopped by adding concentrated H_2_SO_4_, generating a yellow color. The final absorbance in each micro-well was measured by a plate reader spectrophotometer (MultiSkan FC ThermoScientific, Thermofisher Scientific, Waltham, MA, USA) at λ = 450 nm within 10 min from the reaction stopping. The calibration curve for the quantitative analysis was then calculated and the final concentrations of the analytes in samples were then interpolated, considering the dilution factor, when necessary. TRP amounts were reported as μM, whereas 5-HT, KYN and QUIN as ng/mL. The dimensionless ratios 5-HT/TRP, KYN/TRP, QUIN/TRP and QUIN/KYN were also calculated as indices of the TRP metabolic fluxes, after having transformed TRP levels into ng/ml.

### 2.4 Data analyses

All data were presented as the mean ± SD and the mean rank. All biochemical assays were conducted at least in duplicate. For all biological parameters investigated here, a 4-PL non-linear regression analysis was conducted to obtain the respectiive standard calibration curve. In this case, the final concentrations in plasma or serum were interpolated as follows: the STD concentrations of the analyte, reported in the corresponding unit of measure, were log transformed and a semi-log calibration curve (Y vs. logX) was built by means of the 4-PL non-linear regression equation. After interpolating the analyte concentration in unknowns, resulting log values were then retransformed into the non-logarithmic measures (X’= 10^logX^). All calibration curves for biochemical dosages were carried out using the GraphPad Prism software (Version 7.0, San Diego, USA). Due to the limited number of subjects recruited in this study and considering that normality tests and variance homoscedasticity were not respected for some variables in our sample, we applied non-parametric analyses for elaborating our data. A Kruskal-Wallis one-way analysis of variance was used to compare continuous socio-demographic variables, psychometric instrument scores and biochemical parameter concentrations among the three groups, followed by Dunn test for *post-hoc* comparisons. For comparisons of continuous nonparametric variables between the two groups of BD patients, the Mann-Whitney U-test was used. Chi-square tests were performed to compare categorical socio-demographic variables among groups. To evaluate the relationships between biochemical variables themselves and between the psychometric instrument scores, Spearman’s analyses were conducted to obtain the correlation coefficient (r_S_). All the statistical comparisons and correlations were performed using SPSS version 28 IBM Corp. (2021). For all the statistical analyses carried out in this study, the statistical threshold was set at *p* ≤.05, to avoid type II errors.

## 3 Results

The final sample of the present study included 20 BD subjects in the euthymic phase with PTSD (PTSD group), 20 BD subjects experiencing a major depressive episode (DEP group) and 24 healthy controls (CTL group). Sociodemographic and clinical characteristics were reported elsewhere (82). All patients were under a psychopharmacological treatment that was stable with respect to drugs and dosages for at least one month before entering the study, according to the established inclusion criteria. Particularly, thirty-four (85.0%) patients were receiving an antidepressant, 33 (82.5%) a mood stabilizer, 30 (75.0%) an antipsychotic and 24 (60.0%) benzodiazepine. There was no significant difference among the number of patients taking antidepressants (15, 44.1% vs 19, 55,9%; p=0.182), mood stabilizers (14, 42.4% vs 19, 57.6%; p=0.091) and benzodiazepines (10, 41.7% vs 14, 58.3%; p=0.197) in the two subgroups of BD patients (PTSD vs DEP group, respectively). There was instead a significant higher number of subjects taking antipsychotic medications in the DEP group vs. the PTSD one (19, 63.3% vs 11, 36.7%; p=0.011). As shown in **Table 1**, significant differences were found for TRP, 5-HT and QUIN levels, while no difference was reported in KYN levels among groups, despite lower values obtained in PTSD patients. The levels of TRP were significantly lower in the DEP group than in the CTL one. Both PTSD and DEP groups exhibited lower 5-HT levels when compared with CTL group, and DEP group reported lower 5-HT levels than PTSD one. Further, DEP group showed significantly higher QUIN concentrations than the CTL group; QUIN levels in plasma were also higher in PTSD than CTL subjects, but lower than DEP patients, without however reaching the statistical significance.

**Table 1:** Comparison of TRP, its major metabolites and ratios in the subjects’ groups under investigation.

Biological parameters	PTSD (n=20) *Mean ± SD, Mean rank*	DEP (n=20) *Mean ± SD, Mean rank*	CTL (n=24) *Mean ± SD, Mean rank*	H	P	*Post-hoc*
TRP, μM	56.43 ± 17.43, 33.15	44.55 ± 12.61, 21.35	67.17 ± 25.70, 41.25	12.50	.002	*DEP<CTL*
5-HT, ng/mL	4.03 ± 2.32, 25.38	2.00 ± 1.14, 13.14	16.39 ± 6.27, 47.65	39.50	<.001	*DEP, PTSD <CTL;* *DEP<PTSD*
KYN, ng/mL	405.80 ± 124.96, 25.28	508.98 ± 148.95, 37.35	563.58 ± 304.10, 34.48	4.64	.098	*-*
QUIN, ng/mL	80.43 ± 58.29, 31.84	103.24 ± 59.86, 42.42	53.28 ± 26.70, 22.58	12.83	.002	*CTL<DEP*
5-HT/TRP	0.0004 ± 0.0002, 24.10	0.0003 ± 0.0002, 16.11	0.0015 ± 0.0008, 45.18	30.64	<.001	*DEP, PTSD<CTL*
KYN/TRP	0.04 ± 0.02, 27.65	0.06 ± 0.02, 43.60	0.05 ± 0.05, 27.29	10.34	.006	*PTSD, CTL<DEP*
QUIN/TRP	0.008 ± 0.0078, 29.94	0.015 ± 0.012, 44.74	0.005 ± 0.0037, 20.92	19.18	<.001	*PTSD, CTL < DEP*
QUIN/KYN	0.218 ± 0.17 37.45	0.224 ± 0.15 40.89	0.11 ± 0.071 20.42	15.83	<.001	*CTL < PTSD, DEP*

H: Kruskall Wallis significance test; *post hoc*: Dunn’s analysis.

**Figure 2:**
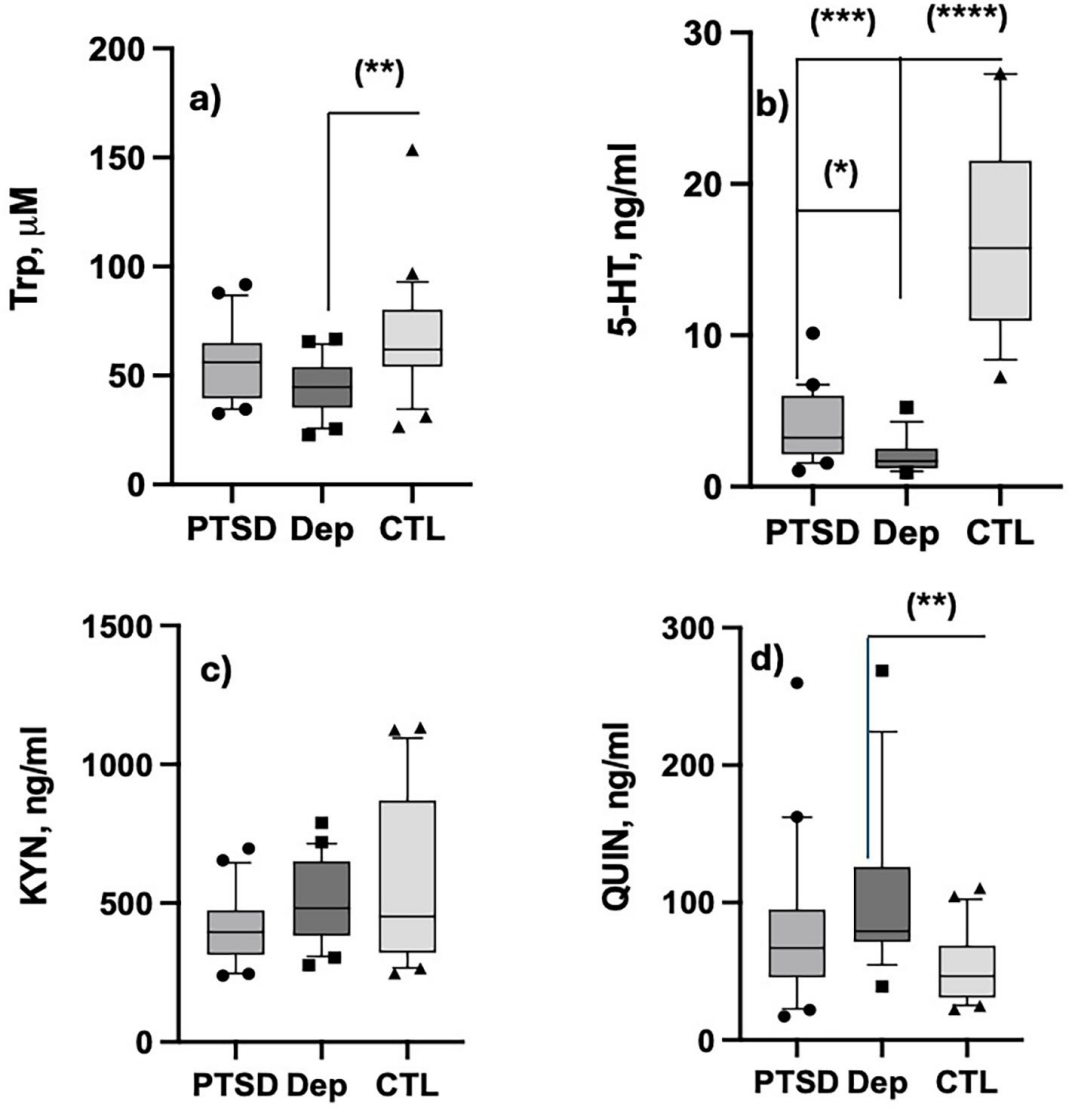
Graphical representation of the results obtained for plasma TRP, 5-HT, KYN and QA presented in **Table 1**. Data are presented as. as boxplot of medians and 10–90 inter quartiles. **(a)** Tryptophan; **(b)** Serotonin; **(c)** Kynurenine; **(d)** Quinolinic acid; Kruskal Wallis significance: (*): p ≤.05; (**): p<.01; (***): p <.001; (****): p <.0001.

**Figure 3:**
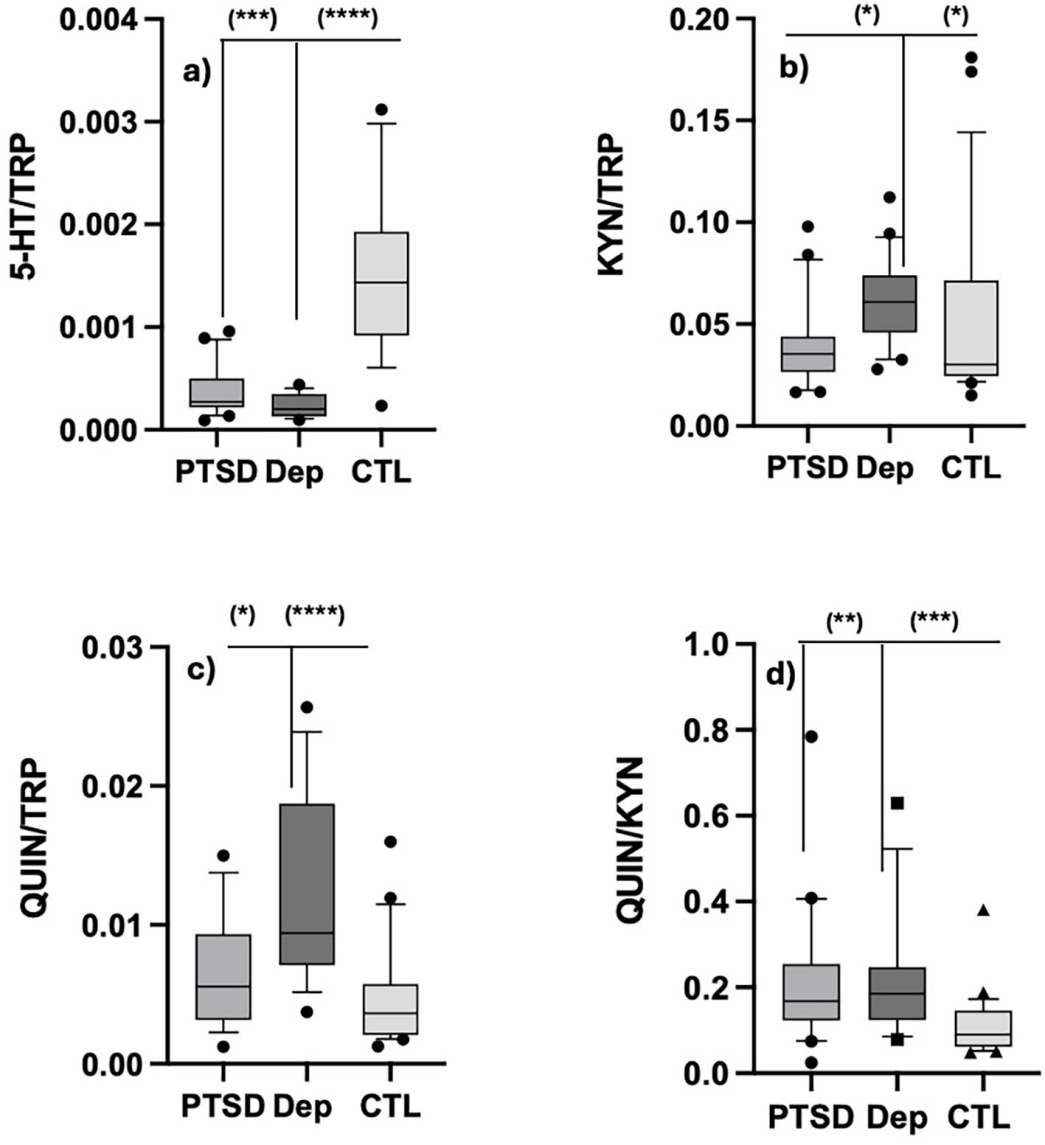
Graphical representation of the results obtained for plasma 5-HT/TRP, KYN/TRP, QUIN/TRP and QUIN/TRP presented in **Table 1**. Data are presented as. as boxplot of medians and 10–90 inter quartiles. **(a)** Serotonin/tryptophan ratio; **(b)** Kynurenine/tryptophan ratio; **(c)** Quinolinic acid/tryptophan ratio; **(d)** Quinolinic acid/kynurenine ratio. Kruskal Wallis significance: (*): p ≤.05; (**): p<.01; (***): p <.001; (****): p <.0001.

To better evaluate the TRP-metabolism fluxes in different subjects, we also compared the between-group differences of the various ratios. The 5-HT/TRP and the QUIN/KYN ratios were significantly lower and higher, respectively, in both PTSD and DEP groups when compared with CTL subjects. Moreover, KYN/TRP and QUIN/TRP ratios were significantly higher in the DEP group only, when compared with the other two groups. These results are also depicted in **Figure 2** (TRP, 5-HT, KYN and QUIN) and **Figure 3** (5-HT/TRP, KYN/TRP, QA/TRP, QA/KYN) as box-blot graphs, together the specific between-group *post hoc* significance obtained.

When evaluating the Spearman correlation coefficient among biochemical parameters in the whole sample, we found that, as shown in **Table 2**, TRP levels were positively correlated with 5-HT, while being negatively correlated to QUIN concentrations and KYN/TRP, QUIN/TRP and QUIN/KYN ratios. Also, 5-HT levels were significantly and positively correlated to 5-HT/TRP ratio, while they were negatively correlated to QUIN, QUIN/TRP and QUIN/KYN ratios. KYN concentrations were positively and negatively correlated, respectively, with KYN/TRP and QUIN/KYN ratios, as well as QUIN levels were positively correlated to KYN/TRP, QUIN/TRP and QUIN/KYN ratios. KYN/TRP ratio was positively correlated to QUIN/TRP ratio.

**Table 2:** Correlations (Spearman r) between biochemical parameters in the whole sample (n = 64).

Biological parameters	TRP, μM	5-HT, ng/mL	KYN, ng/mL	QUIN, ng/mL	5HT/TRP	KYN/TRP	QUIN/TRP	QUIN/KYN
TRP, μM	–	–	–	–	–	–	–	–
5-HT, ng/mL	.353**	–	–	–	–	–	–	–
KYN, ng/mL	-.099	.036	–	–	–	–	–	–
QUIN, ng/mL	-.339**	-.344*	.190	–	–	–	–	–
5-HT/TRP	-.011	.915**	.036	-.249	–	–	–	–
KYN/TRP	-.696**	-.234	.740**	.406**	.012	–	–	–
QUIN/TRP	-.699**	-.439**	.192	.881**	-.196	.619**	–	–
QUIN/KYN	-0.29*	-.375**	-.454***	.759****	-.309*	-.088	.706****	–

Bold Spearman r values represent the significant ones. (****): p<.0001; (***), p<.001; (**): p<.01; (*): p<.05.

We also appraised the possible correlations among biochemical parameter concentrations and the total and domain scores reported on psychometric scales. As described in the method section, for all the scales employed higher scores were associated with a higher impairment in the investigated dimension. These results are presented in **Table 3**, for the HAM-D, YMRS, IES results and MOOD-SR scores. **Table 4** for the TALS-SR and WSAS outcomes. We found that plasma TRP, 5-HT and 5-HT/TRP ratio were significantly and negatively correlated with current HAM-D total score, whereas QUIN concentrations, KYN/TRP, QUIN/TRP and QUIN/KYN ratios were positively correlated to it. The YMRS total score presented a similar correlation profile except for the KYN/TRP ratio. Considering the lifetime MOOD-SR Spectrum questionnaire, only 5-HT plasma levels and the 5-HT/TRP ratio negatively correlated with all domains and total scores of this psychometric instrument. As concerns the KYN-shunt parameters, QUIN/TRP or QUIN/KYN ratios positively correlated with all domains and total scores of MOOD-SR, except for the *Cognition Manic* domain. As regards TRP and QUIN plasma levels *per se*, these parameters were found negatively and positively correlated respectively with some subdomains of MOOD-SR: TRP negatively with *Energy Depressive* and *Rhythmicity* domains, besides the *Total Depressive* score; QUIN positively with *Mood Depressive* and *Mood Energy* domains. As regards the trauma dimension, 5-HT and 5-HT/TRP ratio were uncovered to correlate negatively, while QUIN/TRP and QUIN/KYN ratios positively, with total and all-domain current severity IES-R scores. The TRP plasma levels did not correlate with any of the IES-R domains, while plasma QUIN was positively connected with the IES-R *Hyperarousal* domain only. When exploring correlations between biochemical parameters and lifetime TALS-SR Spectrum, we still found significantly negative correlations between all TALS-SR domain scores, 5-HT levels and 5HT/TRP ratio (except for the *Loss Events* domain). All the other parameters significantly correlated only with some lifetime trauma domains: TRP plasma amounts negatively correlated with *Loss Events*, *Maladaptive coping* and *Personal Characteristics/Risk Factors* domains scores; QUIN and KYN/TRP ratio positively and uniquely with the *Potential Traumatic Events* domain and *Loss Events* domain score, respectively. The QUIN/TRP ratio positively correlated to *Loss Events, Potentially Traumatic Events, Avoidance and numbing* and *Arousal* domains scores. The QUIN/KYN ratio positively correlated instead to *Grief Reaction and Potentially Traumatic Event* domain values.

**Table 3:** Correlations between biochemical parameters, current HAMD, YMRS, IES and lifetime MOOD-SR results in the whole sample (Spearman r, n = 64).

Biological parameters	HAM-D	YMRS	IES-R				MOODS-SR								
	Total score	Total score	Intrusion domain	Avoidance domain	Hyperarousal domain	Total score	Mood depressive domain	Mood manic domain	Energy depressive domain	Energy manic domain	Cognition depressive domain	Cognition manic domain	Rhythmi-city domain	Total depressiv-e score	Total manic score
TRP, μM	-.424**	-.335**	-.205	-.242	-.162	-.191	-.213	-.195	-.351**	-.242	-.208	-.043	-.280*	-.257*	-.185
5-HT, ng/mL	-.792**	-.634**	-.509**	-.496**	-.475**	-.505**	-.635**	-.449**	-.477**	-.496**	-.576**	-.333*	-.472**	-.617**	-.469**
KYN, ng/mL	.065	-.072	-.193	-.212	-.190	-.197	-.006	-.087	.005	-.037	-.082	.006	-.056	-.041	-.048
QUIN, ng/mL	.444**	.382**	.233	.228	.271*	.242	.256*	.217	.271*	.298*	.204	.137	.178	.248	.238
5-HT/TRP	-.678**	-.578**	-.516**	-.486**	-.494**	-.519**	-.619**	-.428**	-.390**	-.462**	-.551**	-.344*	-.405**	-.575**	-.447**
KYN/TRP	.354**	.215	.029	.019	-.019	.006	.130	.078	.261*	.130	.102	.036	.148	.151	.098
QUIN/TRP	.535**	.428**	.251*	.277*	.262*	.261*	.288*	.279*	.354**	.346**	.276*	.160	.306*	.311*	.293*
QUIN/KYN	.451**	.487**	.402**	.427**	.433**	.417**	.329**	.376**	.302**	.385**	.327*	.212	.317**	.372**	.363**

Bold Spearman r values represent the significant ones. (**): p<.01; (*): p ≤.05.

**Table 4:** Correlations between biochemical parameters and lifetime TALS-SR and functional WSAS scores in the whole sample (Spearman r, n = 64).

Biological parameters	TALS-SR									WSAS				
	Loss events domain	Grief reactions domain	Potentially traumatic events domain	Reactions to losses or upsetting events domain	Re-experiencing domain	Avoidance and numbing domain	Malada-ptive coping domain	Arousal domain	Personal characteristics/risk factors domain	Work	Home management	Social leisure activities	Private leisure activities	Close relationships
TRP, μM	-.302*	-.143	-.174	-.161	-.185	-.228	-.288*	-.147	-.304*	-.319**	-.317*	-.247*	-.276*	-.406**
5-HT, ng/mL	-.335*	-.443**	-.570**	-.436**	-.373**	-.402**	-.333*	-.468**	-.422**	-.670**	-.669**	-.674**	-.669**	-.646**
KYN, ng/mL	.043	.017	-.177	.089	.087	.048	-.040	.002	-.185	-.148	-.081	-.072	-.006	-.061
QUIN, ng/mL	.197	.193	.271*	.007	.127	.219	.091	.236	.155	.308*	.359**	.394**	.394**	.367**
5-HT/TRP	-.232	-.462**	-560**	-.430**	-.360**	-.376**	-.292*	-.475**	-.349**	-.603**	-.595**	-.639**	-.630**	-.537**
KYN/TRP	.288*	.131	.014	.192	.220	.209	.163	.136	.061	.137	.171	.125	.191	.209
QUIN/TRP	.257*	.231	.256*	.125	.177	.284*	.210	.263*	.237	.384**	.406**	.428**	.436**	.457**
QUIN/KYN	.227	.257*	.411**	.08	.118	0.216	.205	.234	.358**	.493**	.470**	.459**	.453**	.474**

Bold Spearman r values represent the significant ones. (**): p<.01; (*): p<.05.

We then evaluated the correlations between biochemical parameters and the WSAS, which explores the negative impact of symptoms on different areas of functioning, such as work and social activities. Significantly negative correlations emerged among the levels of TRP, 5-HT, 5-HT/TRP ratio, and almost all WSAS items scores. As well, significantly positive correlations were shown among QUIN, QUIN/TRP and QUIN/KYN ratios and all WSAS items scores. Plasma levels of KYN were found unrelated to all the mood and trauma psychometric tools employed herein.

## 4 Discussion

To the best of our knowledge, this is the first study investigating some of the main metabolic substrates deriving from the essential amino acid TRP in relation to a mood disorder diagnosis such as BD through specific stages/states of the disease, i.e., precisely, during a major depressive episode (DEP group) or the exclusive presence of PTSD symptoms (PTSD group), compared with healthy controls. A recent metabolomic work has reported different biological substrates in BD patients with mania, depression or mixed episodes (83), without considering subjects concomitantly affected by PTSD.

Our results are relevant in that they preliminary show the presence of low 5-HT with distinct patterns of TRP amounts and KYN metabolism in the two psychiatric conditions within BD, which also differ from the control group. Primarily, we observed a significant and substantial reduction in plasma 5-HT levels in both the DEP- and PTSD-groups compared to controls. Furthermore, 5-HT concentrations were also significantly lower in DEP- than in PTSD-patients. This more pronounced depletion of plasma 5-HT in the DEP-group supports a prominent role of the monoamine in this mental dimension (84). The classical monoamine theory of depression, formulated in the early ‘60s argues that mood depressed symptoms result from a kind of “biochemical lesion” in the CNS, generated by an unbalance between 5-HT and catecholamine neurotransmission (85), and/or due to a prevalent impairment within the 5-HT system (86, 87). Starting from this concept, the amounts of 5-HT in the bloodstream, along with the appraise of platelet 5-HT molecular targets such as platelet serotonin transporter (SERT), have been measured for years as indicators of the status, trait, and response to treatment of mood disorders (88–101). Among these studies, one of the earliest reported that the free 5HT pool detected in human plasma can be found reduced in drug-free melancholic patients without TRP decrease and without responding to the tricyclic antidepressant clomipramine (88), suggesting that low 5-HT in plasma found in this work would rather be a trait marker of depression more specifically belonging to 5-HT system. Other subsequent investigations showed instead results rather congruent with changes of circulating 5-HT in respect to applied drug therapies. A prospective study conducted in baseline drug-naive patients showed an increase in both 5-HT and antidepressant levels in plasma and platelets, following administration of the SSRI fluoxetine (96). In other papers, plasma/serum levels of 5-HT were found increased in both depressed and manic patients after SSRI and lithium therapies, accordingly to the drug action on 5-HT system (90, 97). However, these findings have not always been replicated (99, 100, 102), and in some of these works, antidepressants were also found to reduce plasma/serum levels of 5-HT, suggesting a complex influence of therapy on bloodstream 5-HT, involving several factors such as genetic heterogeneity, length and level of drug exposure, as well as disease severity and chronicity characteristics.

On the other hand, the monoamine theory has been revised over the years, suggesting more complex causative pictures of depression and mood disorders, involving inflammation-immune mediators (103–110). In agreement with a comprehensive hypothesis of mood disorders and depression, there is increasing conviction that these are multifaceted and heterogeneous mental conditions requiring multidimensional investigative approaches, in which various neurochemical, neuroendocrine and immune/inflammatory pathogenetic factors are assumed to be dynamically interconnected, a viewpoint that may explain the apparently conflicting neurobiological hypotheses formulated so far (104, 111, 112). This concept supports the presence of heterogenous molecular signatures underlining single mental diagnostic dimensions as depression, following somehow the spectrum concept (71). A dimensional approach to depression comprises even subthreshold or atypical symptoms of the illness, including somatic comorbidities or somatic premorbid conditions. Considering such a varied model, the evaluation of biomarkers in distinct groups of bipolar patients without overlapping depression and PTSD symptoms, as in the present research, is relevant and pioneering, while the current results still support the concept that abnormal 5-HT represents a core biochemical feature of depression. It should be then underscored that 5-HT is not only a brain neurotransmitter but also one of the main initiators of the inflammatory response in the body, acting as a vasoactive player, released by platelets and leukocytes, along with other biogenic amines such as histamine, peptides, eicosanoids, proinflammatory cytokines, and acute phase proteins (113, 114): essentially, 5-HT holds the ability to modulate both behavioral and inflammatory defense responses related to depression (114, 115). Plasma concentrations of 5HT are the result of a fine-tuned equilibrium between the platelet uptake, peripheral synthesis and release of this biogenic amine into the bloodstream mostly by enterochromaffin (EC) cells and other cell sources (116, 117). This equilibrium can considerably change during the stress response through the function of the brain-gut axis (118, 119). Precisely, variations in brain-periphery cross-talks that occur during allostasis processes and adaptive responses to external events may alter peripheral-circulating 5-HT contents, by also changing BBB transport abilities and shifting TRP fates towards 5-HT or KYN metabolites, with the cooperation of the gut microbiota (120).

Since 5-HT cannot easily pass across the blood-brain barrier (BBB), these networks can be particularly critical for conditioning5-HT amounts and reserve in the brain, which depend upon the plasma levels of its essential amino acid precursor introduced with diet. Our findings of reduced plasma 5-HT in BD patients with PTSD vs healthy controls, even if in a lesser extent than in DEP patients, sustain a 5-HT dysregulation in PTSD too. This monoamine exerts indeed a key role in the regulation of fear and stress in specific brain areas such as amygdala and hippocampus (121, 122). Concurrently, present observation of a 5-HT deficiency greater in major depressive episodes than PTSD may corroborate the empirical observation of partial/incomplete response to serotonergic treatments that more frequently characterizes trauma symptoms than depressive ones (65, 66, 123). It is tempting to hypothesize that low circulating levels of 5-HT would be related to serotonergic imbalances that occur in bipolar depression primarily due to chronicity aspects of the illness and drug resistance (genetic and/or epigenetic), while they would be more dependent to alterations in neuroendocrine pathways of stress in bipolar patients presenting “event-responsive” trauma symptoms. Nevertheless, from these results it also emerged that plasma 5-HT alone is not a full-specific marker of depression vs. PTSD. It is rather the measure of TRP, KYN, QUIN levels and their ratios in the context of low 5-HT levels that have been able to reveal different TRP trajectories among the two groups of BD patients under investigation. The measure of TRP and KYN shunt metabolites also permitted to appraise the degree of TRP biotransformation into the KYN route, which is induced by the stress response and inflammatory molecular patterns, and its concomitant effect on 5-HT availability within the body (124). Interesting results were obtained regarding TRP and KYN metabolites, which seem in agreement with the concept that stress and/or immune/inflammatory responses may differentially influence TRP levels and fates in distinct mental dimensions, including depression. Plasma TRP was found diminished in DEP subjects only vs. controls, although a lower but not significant median was found in PTSD patients. Furthermore, which is quite surprising, plasma KYN was found unchanged in the three groups under investigation, being lower in the PTSD one without attaining the statistical threshold. The KYN/TRP ratio resulted instead moderately increased in DEP vs. PTSD patients, as well as vs. healthy subjects. Complementary results to TRP were observed for QUIN in plasma and its ratios: QUIN was found significantly increased in patients with depression vs controls only, whereas the PTSD group showed a higher but not significant QUIN median. Moreover, the QUIN/TRP ratio was significantly higher in DEP patients vs. both the PTSD and control subjects while the QUIN/KYN one being significantly raised in both DEP and PTSD subjects in comparison with healthy volunteers. To understand these intricated results, several aspects should be considered. Firstly, it should be mentioned that the plasma amounts of TRP, the precursor of 5-HT and KYN pathways, undergo a tight and subtle regulation. The concentration of this essential amino acid in plasma ranges commonly from about 40 to 80 µM in healthy subjects, with mean values of about 60 µM (125). Plasma levels of TRP, which is both a glucogenic and ketogenic amino acid, depend not only on diet composition and the hormones insulin and glucagon, but, as with other protein amino acids including non-essential ones, are additionally controlled by stress-related signal molecules such as catecholamines and cortisol. They therefore depend on the balance between the stress response defined by “fight-or-flight” and/or HPA activities and the molecular “rest-and-digest” paradigm. Insulin promotes the uptake of amino acids, including TRP, in tissues and cells for protein synthesis after meals; conversely, glucagon, catecholamines and cortisol can attenuate protein synthesis while enhancing proteolysis and TRP release from myocytes and other cells, thus increasing the amino acid concentration in plasma to promote gluconeogenesis for boosting energy during the fasting phase and/or stress response. Under these circumstances, protein synthesis is rather directed towards the expression of specific adaptive protein patterns. Hormones are also responsible of the availability of TRP to the CNS for the synthesis of 5-HT: the TRP carrier located on the BBB is insulin-dependent, since insulin promotes the uptake of competing LNAAs, such as valine, leucine and isoleucine, into muscles favoring, therefore, the passage of TRP into the brain by common transporters as the LAT-1 carrier (126–128). Lipolysis and factors regulating the plasma levels of non-esterified fatty acids (NEFAs) also play a role on TRP passage across the BBB, exerting competition for LAT carriers but also promoting, under specific conditions as physical activity, the shift from the albumin-bound TRP to the free form (129, 130). Beside the above-mentioned hormonal and neuroendocrine mechanisms, induction of KYN shunt enzymes is also capable of varying plasma levels of TRP by competing with TPH activity. Precisely, TDO activity has been found to primarily affect plasma levels of TRP, unlike IDO, which is not specific for TRP (131). Rather, the activation of IDO may be relevant for its action in the formation of the key metabolite KYN, being influenced by the reactive status of immune and inflammatory cells (131, 132); conversely, TDO can be upregulated by increased HPA function and catecholamine release (132). Thus, in DEP patients there could be a decrease of TRP due to abnormal HPA axis functions and metabolic hormone signaling, inducing TDO activity to compete for TPH and 5-HT biosynthesis, without fully recovering this amino acid in plasma. Mechanisms controlling TRP and 5-HT amounts are presumably dysregulated, resulting in low TRP and even lower 5-HT at fasting. In this respect, we mention here that a disturbed glycogen synthase kinase 3 (GSK3) activity, a molecular target of insulin and insulin-like hormones, has been found altered in mood disorders (56, 133).

As concerns the finding of unchanged KYN levels among the three groups under investigations, this could be explained by the central position of KYN in the whole shunt, being the starting point of different routes and destinies: KYN levels can depend on the relative fueling of each of these sub-branches the one to the other. Moreover, accordingly to the molecular model supporting the relationship between altered stress responses and abnormal immune and inflammatory activation, increased plasma QUIN amounts within the DEP group only, suggest that 5-HT availability is also restricted by the concomitant upregulation of IDO and crucial enzymes in the KYN shunt of these subjects, such as downstream Kynurenine Monooxygenase (KMO) and other enzymes conveying this pathway preferentially towards the NAD^+^ branch and QUIN accrual (43–45, 134). This hypothesis is supported by the higher levels of QUIN in plasma, together increased KYN/TRP, QUIN/TRP and QUIN/KYN ratios, found in depressed BD patients. Such results can have also clinical relevance for monitoring depressed patients under therapy, since low plasma levels of 5-HT and increased plasma KYN/TRP or QUIN/TRP ratios have been related to the tendency at resistance towards antidepressant pharmacological interventions (135). The PTSD group showed a quite different TRP metabolism profile from the DEP one, with just the QUIN/KYN ratio being significantly increased in respect to controls. This difference between traumatized and depressed bipolar patients, in the presence of reduced plasma 5-HT in both groups, is particularly surprising, since inflammation, cytokine release and ROS, previously found altered in PTSD, are all factors concurrent to the increase of KYN metabolites, including KYN itself (51, 124, 136). We thus supposed different stress responses between these subjects, e.g., the presence of counterregulatory actions in PTSD that are instead attenuated or lacking in DEP subjects. For instance, in PTSD patients, at least in a part of them, there could be enhanced basal proteolysis resulting from additional cortisol release counteracting TRP depletion, implying a relatively dampened glucocorticoid response in bipolar depression compared with trauma clinical features. Intriguingly, the QUIN/KYN ratio was significantly higher in PTSD without a significant increase of the KYN/TRP ratio, an index of IDO activation. It could happen that IDO is not induced in these patients as it was in the DEP group, or, as well, this enzyme activation could be counteracted and somewhat “hidden” in the PTSD one, as also observed by other authors in KYN metabolism of autism (137). In this regard, it should be remembered that, if KYN levels did not differ among DEP, PTSD and CTL subjects, on the other hand the obtained KYN median was the lowest in the plasma of PTSD patients among the three groups of tested subjects. Some authors have found that subjects with mood disorders and BD can have lower plasma levels of KYN (57, 138, 139). In euthymic BD patients with PTSD, the KYN pathway might be less oriented toward the formation of the downstream metabolite QUIN, unlike what presumably occurs in bipolar depression: rather, there might prevalently be, in these subjects, a KYN biotransformation activating in parallel other enzymatic activities such as Kynureninase (Kynurenine hydrolase, KYNase), which might shift the pathway to other branches, along with only a moderate upregulation of main downstream enzymes as KMO. It is interesting to note that KYNase can compete directly with KMO for KYN, giving rise to AA, while also being an enzyme activated by KMO products (35), a metabolic pattern that suggests possible unbalanced reciprocal induction/expression of these two enzyme activities in PTSD. The enzyme Kynurenine aminotransferase (KAT) could be involved too, for instance in the production of KYNA and XA, this last being a KYN shunt metabolite also endowed with CNS activities and signaling properties (140, 141). Alternatively, the increased QUIN/KYN ratio in PTSD could also derive from a different distribution of KYN in body compartments, such as increased passage of this molecule through the BBB or its recruitment for binding to the aryl hydrocarbon receptor (Ahr) that promotes ROS modulation, all of which could potentially occur during the induction of cytokine release and inflammation present in these patients (142). Briefly, KYN plasma levels in PTSD could be relatively lower in respect to the QUIN ones for multiple reasons, while being influenced by other unknown factors promoting a selective induction of alternative KYN-shunt enzymes.

At the same time, the Spearman correlations between biochemical variables in all subjects widely reflected the pathophysiological fundamentals of TRP metabolism. Indeed, 5-HT levels were positively correlated to the concentrations of TRP, its precursor in the indole-conservating pathway. Also, 5-HT and TRP levels were negatively correlated with QUIN, the metabolic product of the KYN shunt of TRP. These relationships thus overall support the induction of TRP metabolism towards the KYN path and QUIN branch in contrast to 5-HT formation in the whole examined group of subjects.

**Figure 4:**
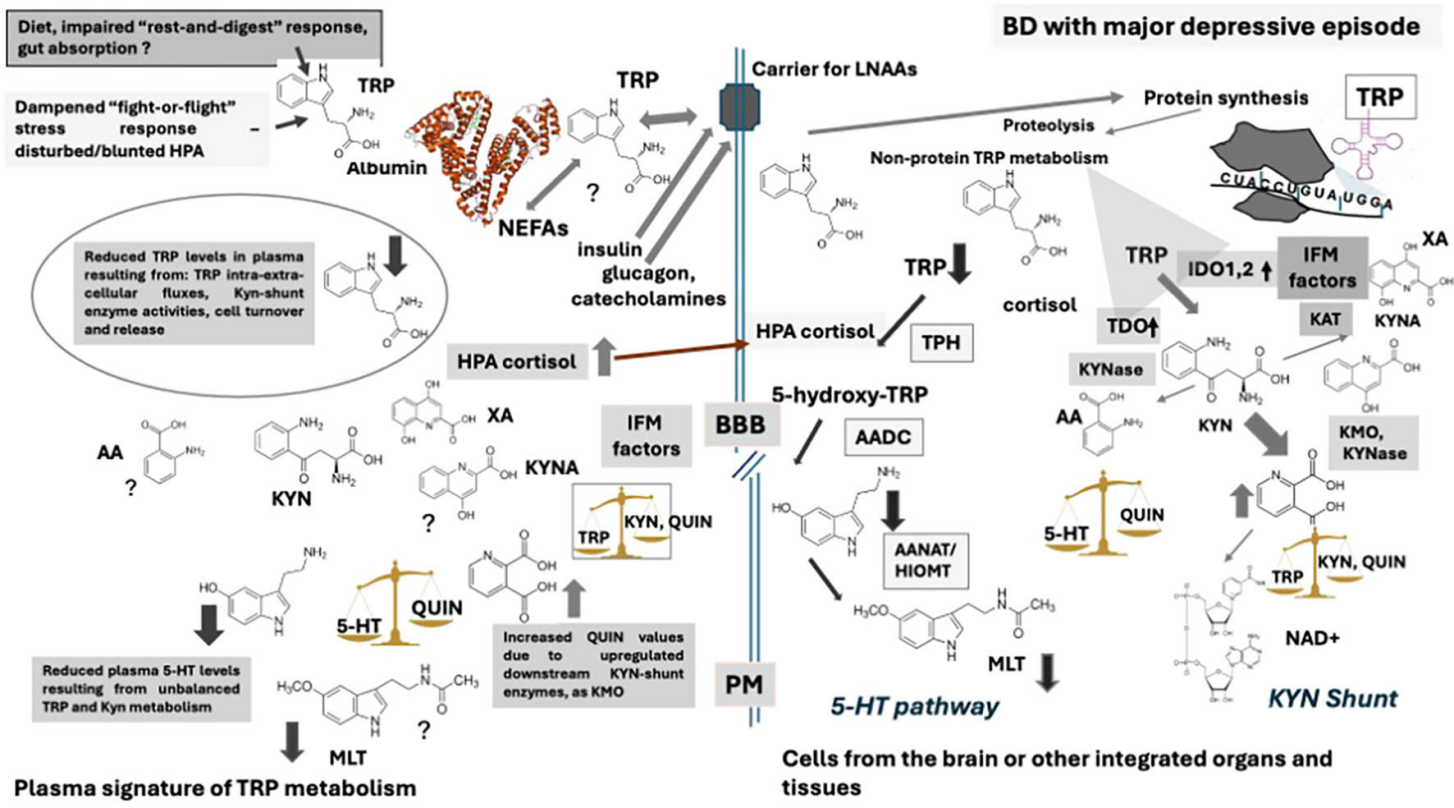
Hypotheses based on results obtained in plasma of BD patients with major depressive episode. Light and dark gray arrows represent increased and decreased levels/activity respectively, while arrow thickness the degree of variation. TRP: Tryptophan; 5-HT: Serotonin; MLT: Melatonin; KYN: Kynurenine; QUIN: Quinolinic acid; KYNA: Kynurenic acid; AA: Anthranilic acid; XA: Xanthurenic acid; NEFAs: Non-esterified fatty acids; LNAAs: Large-neutral amino acids; IFM: inflammatory factors; TPH: Tryptophan hydroxylase; AADC: Aromatic-amino acid decarboxylase; ANAT: Aryl-alkyl-amino-*N*-acetyl transferase; HIOMT: Hydroxy-indole-*O*-methyl transferase; TDO: Tryptophan 2,3 dioxygenase; IDO: Indole 2,3 dioxygenase; KAT: Kynurenine aminotransferase; KYNase: Kynurenine hydrolase; KMO: Kynurenine monooxygenase; BBB: Blood-brain barrier; PM: Plasma membrane.

**Figure 5:**
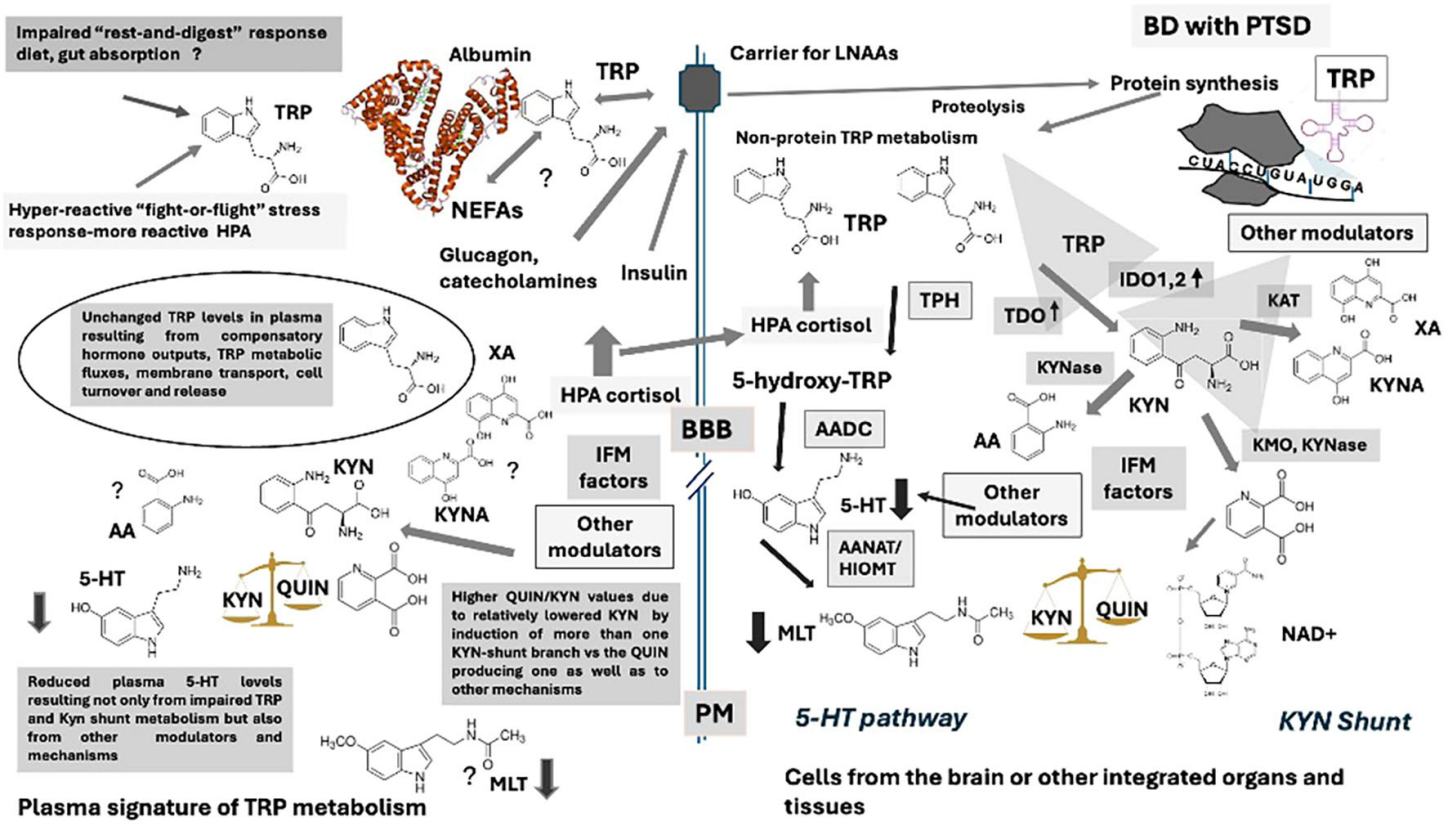
Hypotheses based on results obtained in plasma of BD patients with PTSD symptoms when eutymic. Light and dark gray arrows represent increased and decreased levels/activity respectively, while arrow thickness the degree of variation. TRP, Tryptophan; 5-HT, Serotonin; MLT, Melatonin; KYN, Kynurenine; QUIN, Quinolinic acid; KYNA, Kynurenic acid; AA, Anthranilic acid; XA, Xanthurenic acid; NEFAs, Non-esteri ed fatty acids; LNAAs, Large-neutral amino acids; IFM, in ammatory factors; TPH, Tryptophan hydroxylase; AADC, Aromatic-amino acid decarboxylase; ANAT, Aryl-alkyl-amino-N-acetyl transferase; HIOMT, Hydroxy-indole-O-methyl transferase; TDO, Tryptophan 2,3 dioxygenase; IDO, Indole 2,3 dioxygenase; KAT, Kynurenine aminotransferase; KYNase, Kynurenine hydrolase; KMO, Kynurenine monooxygenase; BBB, Blood-brain barrier; PM, Plasma membrane.

Aside, correlations among biochemical parameters and psychometric variables revealed additional differences among DEP and PTSD patients. These analyses showed that TRP levels negatively correlated with the actual gravity of depressive and manic symptoms, evaluated by means of the HAM-D and YMRS respectively, with some domains of the lifetime dimensional mood spectrum symptomatology assessed by MOODS-SR (particularly *Energy Depressive*, *Rhythmicity* and *Total Depressive score*) and with the impairment in the several areas of subject’s functioning measured by means of the WSAS scale. Besides, 5-HT levels negatively correlated with all scales, e.g. the actual severity of depressive symptoms, evaluated by the HAM-D total score, as well as with the actual severity of manic symptoms, appraised by the YMRS total score, with the lifetime dimensional mood spectrum symptomatology assessed by MOODS-SR and with the impairment in the several areas of subject’s social and personal activities measured by means of the WSAS scale. Remarkably, unlike TRP levels, 5-HT concentrations also negatively correlated with the actual severity of PTSD symptoms, assessed by the IES-R scale, and with the dimensional symptomatology of the PTSD spectrum across the lifespan, measured by the TALS-SR. This last finding suggests a reduction of 5-HT in the two conditions correlated with the specific current and lifetime symptom-severity scales (mood or trauma). Almost a similar profile was reported for the 5-HT/TRP ratio. The observed correlations of both TRP and 5-HT levels with the HAM-D total score can support the more pronounced 5-HT depletion due to TRP reduction in mood disorders, particularly major depressive episode, than in PTSD, since TRP did not correlate with the IES-R results. The 5-HT correlations obtained with the actual severity symptoms of trauma might also support the role of this monoamine in PTSD, presumably under the control of different neurobiological substrate and inflammatory patterns in respect to major depressive episode, as for instance, those produced by alterations of other neurotransmitter mechanisms concurrent to 5-HT impairment, such as glutamate and GABA markers/signaling (143). In fact, unlike TRP, QUIN/TRP and QUIN/KYN were found positively related with the IES-R scales, indicating independent IDO activation from TRP regulation in PTSD. It is noteworthy that QUIN levels positively correlated with the global function impairment, highlighting the possible neurotoxic role of the accumulation of this metabolite. The TRP-deriving QUIN are thought to promote NMDA transmission, in specific brain regions such as the striatum and the hippocampus (44, 54), acting as a glutamatergic excitatory compound produced in response to inflammation (44, 45). As well, QUIN has been reported to activate NMDA receptors, distributed in specific brain regions such as the striatum and the hippocampus (45, 54). The physiological role of this molecule has been linked to its action as a glutamatergic partial agonist under specific conditions as well as to the necessity to increase cell energy through the raise of NAD^+^ levels (45, 54). This substrate has been also linked to lymphocyte differentiation, clonal selection and immunotolerance (144), exerting the function to prevent autoimmune responses during the activation of inflammatory networks. On the other hand, QUIN, similarly to other KYN-shunt downstream substrates as 3-hydroxykynurenine (3HKYN), is a well-known inducer of free radicals under uncontrolled contingencies, thus increasing oxidative stress, ROS production, together the decrease of antioxidant resources as glutathione and SOD activity (44, 45, 54). It has been reported, for instance, that the excess of QUIN is linked to intracellular calcium imbalance and neural apoptosis (145, 146). Our data pointed out a positive correlation between KYN/TRP or QUIN/TRP ratios and the gravity of depressive symptoms, supporting those data reporting increased QUIN and KYN fluxes associated with severity of depression and presence of suicide thoughts/attempts (61, 62). These findings suggest that depression in bipolar disorder, which is commonly relapsing, may feature alterations in KYN metabolic pathways that converge on attenuated 5-HT networks, whereas PTSD would likely be defined by additional, distinct and as yet unknown molecular variation linked to inflammation leading to reduced levels of this monoamine in the bloodstream, albeit to a milder degree (45). Substantially, this hypothesis further supports that low 5-HT levels would underscore diverse pathophysiological and clinical significance in depression and PTSD. **Figures 4** and **5** depict diagrams summarizing our results with respect to some presumed trajectories of TRP pathways in the different BD conditions examined here, **Figure 4**- BD with major depressive episode and **Figure 5**-BD with PTSD symptoms, highlighting the possible hypothetical most relevant influencing factors on these molecular configurations.

This study has some limitations that should be mentioned, and which may affect the generalization of results. First, it was conducted following an observational, cross-sectional experimental design in small subgroups of subjects. As afore reported, subjects were prevalently assessed under pharmacological treatment, a stable condition at the time of the investigation, that could have an impact, albeit putatively modest, on results. The significant prevalence of patients taking antipsychotics in the DEP group could have influenced biochemical differences between DEP and PTSD patients, but currently there is no direct support to this statement. Some studies, indeed, imply few effects of these drugs on the levels of TRP and TRP metabolites, and these were essentially conducted on patients with a full-blown psychotic spectrum disorder (147–152). In any case, antipsychotics were mainly administered in our patients for their mood stabilizer and/or antidepressant properties, at lower dosages than those indicated for patients with a diagnosis of psychotic spectrum disorder. Other limits are the lack of an additional comparative group composed by euthymic BD patients without comorbidities, as well as the incomplete recording of data on the nutritional status, smoking and physical activity of subjects enrolled in the survey, which can potentially bias the results.

## 5 Conclusions

Despite its inherent limitations, this study supports the usefulness of implementing TRP metabolism biomarkers in BD, PTSD, and depression. Under the present experimental conditions, BD patients with low 5-HT levels and severe disease burden were distinguished based on different KYN metabolism profiles/trajectories in the depressive phase, compared to those who were euthymic but had post-traumatic symptoms, as well as compared to controls. “Classic” alterations in the KYN pathway, linked to TRP and 5-HT, appear to be more related to depression than to PTSD. In light to present results, other pharmacological targets than those acting on 5-HT metabolism as SSRIs could be suggested for PTSD, including specific inflammatory molecular patterns, players in the stress response and HPA axis, as well as ROS modulating agents. Clarifying and further investigating these targets, including through the evaluation of other biological parameters and other KYN shunt products by means of longitudinal investigations, will make it possible to obtain biochemical signatures in plasma with respect to the chronicity of the disease and comorbidities (153). This will possibly contribute at identifying long- or short-term profiles, allowing a deep patient monitoring, and, on the one hand, achieving new therapeutic perspectives for the treatment of PTSD while, on the other, counteracting resistance to therapy in bipolar depression.

## Data Availability

The raw data supporting the conclusions of this article will be made available by the authors, without undue reservation.
